# Eggshell membrane powder lowers plasma triglyceride and liver total cholesterol by modulating gut microbiota and accelerating lipid metabolism in high‐fat diet‐fed mice

**DOI:** 10.1002/fsn3.1545

**Published:** 2020-04-05

**Authors:** Nurul Shazini Ramli, Huijuan Jia, Ayumu Sekine, Weida Lyu, Kyohei Furukawa, Kenji Saito, Yukio Hasebe, Hisanori Kato

**Affiliations:** ^1^ Graduate School of Agricultural and Life Sciences The University of Tokyo Tokyo Japan; ^2^ Department of Food Science, Faculty of Food Science and Technology Universiti Putra Malaysia Serdang Malaysia; ^3^ ALMADO Inc. Tokyo Japan

**Keywords:** cholesterol, eggshell membrane, gut microbiota, high‐fat diet, lipid metabolism, triglycerides

## Abstract

Obesity is a major global lifestyle disorder associated with gut microbiota. The health benefits of eggshell membrane (ESM) have been shown in previous reports, particularly as regards gut microbiota composition. Here, we investigated whether ESM improves lipid metabolism and alters gut microbiota in high‐fat diet‐fed mice. A total of 20 C57BL/6J mice aged 6 weeks were given either a control diet (CON), high‐fat diet (HFD), or high‐fat diet + 8% ESM powder (HESM) for 20 weeks. ESM supplementation in HFD‐fed mice reduced plasma triglycerides (TG) and liver total cholesterol (TC) and upregulated the expression of lipid metabolism genes carnitine palmitoyltransferase 1A and suppressor of cytokine signaling 2. Microbiota analysis showed increased relative abundance of the anti‐obesity bacterium, *Lactobacillus reuteri*, at 4, 12, and 16 weeks and reduced the abundance of inflammation‐related *Blautia hydrogenotrophica*, *Roseburia faecis*, and *Ruminococcus callidus* at 12 and 20 weeks. ESM‐supplemented mice had increased cecal isobutyrate, negatively correlated with *B. hydrogenotrophica* and *Parabacteroides goldsteinii* abundance. The results indicate that ESM supplementation in HFD‐fed mice reduced plasma TG and liver TC, possibly through alteration of lipid metabolism gene expression and gut microbiota composition, suggesting that ESM may be effective in obesity management.

## Graphical abstract

Eggshell membrane (ESM) supplementation for 20 weeks in HFD‐fed mice reduced plasma triglycerides (TG) and liver total cholesterol (TC). The upregulated gene expression of lipid metabolism genes carnitine palmitoyltransferase 1A and suppressor of cytokine signaling 2 suggest that lipid metabolism might be accelerated to increase TG production and clearance rates, as well as increase liver cholesterol excretion. Microbiota analysis showed increased relative abundance of the anti‐obesity bacterium and reduced abundance of inflammation‐related bacterium, which were negatively correlated with cecal isobutyrate levels.

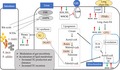

## 1 INTRODUCTION

Over recent decades, obesity has become the major cause of mortality and morbidity worldwide. Obesity is multifactorial in origin and associated with an increased disease burden, particularly diabetes mellitus, ischemic heart diseases, and certain types of cancer (Min, Zhao, Slivka, & Wang, 2018). Mounting evidence has shown a correlation between the gut microbiota and the development of metabolic disorders including obesity. Distinct microbial compositions have been identified in obese and healthy individuals (Hou et al., 2017). Indeed, changes in microbial community structures have been shown to strongly influence the serum metabolome, leading to insulin resistance in the non‐diabetic population (Pedersen et al., 2016). These effects could be related to microbial metabolism products, which act as signaling molecules and directly influence the function of the small intestine, liver, adipose tissue, and the brain (Aleman et al., 2018).

Several studies suggest that dietary intervention containing indigestible food components may stimulate an increase in the relative abundance of certain microbes and subsequently improve metabolic status (Hoving et al., 2018). In addition, the occurrence of certain bacterial species during dietary intervention, such as *Lactobacillus* spp., positively affects insulin sensitivity and reduces plasma cholesterol levels in obese mice (Park et al., 2017). A recent study involving hyperlipidaemia patients receiving rosuvastatin treatment showed that the hypolipidemic effect was associated with a variation in gut microbiota composition (Liu et al., 2018).

Eggshell membrane (ESM) is regarded as industrial waste and is often discarded after egg processing. ESM is rich in fibrous protein and contains moderate amounts of collagen, hyaluronic acid, chondroitin sulfate, and glucosamine (Ruff, DeVore, Leu, & Robinson, 2009). Previously, we have demonstrated the ability of 8% ESM to attenuate microbial dysbiosis and intestinal inflammation in an animal model of inflammatory bowel disease (Jia et al., 2017). As ESM is rich in low digestible protein and might possess physiological properties similar to that of dietary fiber, we investigated the anti‐obesity effects of ESM and its association with the gut microbiota.

## 2 MATERIALS AND METHODS

### 2.1 Animals and dietary treatment

The experimental group consisted of 20 C57BL6J male mice, aged 6 weeks supplied by Charles River Japan. The mice were individually housed in cages in a temperature controlled (23 ± 2°C), 12‐hr light–dark cycle environment with a relative humidity of 50%–60%. The experimental animals were acclimatized for 3 days and then randomly divided into three groups based on their diet: control diet (CON: *n* = 7), high‐fat diet (containing 45% fat; HFD: *n* = 7), or high‐fat diet + 8% ESM powder (HESM: *n* = 6) for 20 weeks. Feces were collected every 4 weeks for microbiota analysis. The study protocols were approved by the Animal Care and Use Committee of the University of Tokyo (Approval No. P17‐143). All procedures were conducted according to the relevant rules and regulations.

### 2.2 Dosage regimen

The HESM diet was supplemented with 8% ESM as previously described by Jia et al. (2017). As the digestibility of ESM is approximately 46%, the HESM diet was adjusted with cornstarch and casein to maintain the caloric and protein balance (Table S1). The daily intake of ESM was approximately 0.24 g per animal, as the food intake of mice is approximately 3 g/day. This is equivalent to an estimated human daily dose of 53 g for a 60 kg individual, which is high but can be consumed via supplements, cookies, and noodles.

### 2.3 Blood collection and tissue harvesting

Terminal anesthesia was performed via intraperitoneal injection of pentobarbital sodium. Blood was collected in heparinized tubes and immediately centrifuged at 1,000 *g* at 4°C for 15 min to obtain plasma for further analysis. The liver, adipose tissue, and cecal content were collected and stored at −80°C prior to analysis.

### 2.4 Biochemical assays

Hepatic lipids were extracted using chloroform–methanol (2:1, v/v) according to the method of Folch et al. (Folch, Lees, & Sloane Stanley, 1957). We next determined the concentrations of hepatic and plasma triglycerides (TG), total cholesterol (TC), nonesterified fatty acids (NEFA), and phospholipids (PL) according to the manufacturer‐provided instructions using kits (Wako).

### 2.5 RNA extraction and real‐time RT‐PCR

Total RNA was extracted from the frozen liver using TRIzol reagent (Invitrogen) and an RNA Isolation Kit (NucleoSpin RNA II; Macherey Nagel), as described by Jia et al. (2017). The expression values of specific genes were normalized against the expression level of glyceraldehyde‐3‐phosphate dehydrogenase (*Gapdh*) in the liver. The primer sequences for the genes used in the present study are detailed in Table S2.

### 2.6 Fecal and cecal DNA extraction and metagenomic analysis

Fecal and cecal DNA extraction was conducted using the QIAamp Stool Mini Kit (Qiagen) according to the manufacturer's instructions and the method of Jia et al. (2017). Variable regions 3 and 4 of the 16S rRNA gene were amplified using primers (5′‐CCTACGGGNGGCWGCAG‐3′ and 5′‐GACTACHVGGGTATCTAATCC‐3′) modified to incorporate Illumina adapters and barcode sequences for subsequent sequencing. Next, we performed library size and quantification analysis using the Agilent 2100 Bioanalyzer (Agilent Technologies) and all libraries were pooled on a single Illumina MiSeq run (MiSeq Reagent Kit V3, 600 cycles; Illumina) based on the manufacturer's instructions and protocols. The overlapping paired‐end reads were combined using the Quantitative Insights Into Microbial Ecology (QIIME v.1.8.3) software (http://www.qiime.org). The resulting sequences were clustered into operational taxonomic units (OTUs), with a threshold of 97% pairwise identity. The representative sequences were classified by the Ribosomal Database project (RDP) classifier in QIIME based on the Greengenes OTU database. Principal coordinate analysis (PCoA) was used to determine the distance between samples.

### 2.7 Cecal SCFA analysis at 20 weeks

The cecal SCFA concentration was determined using ion‐exclusion HPLC according to the method of Tsukahara et al. (2014). The collected cecal contents (0.05 g) were mixed with distilled water (0.1 ml) and 12% perchloric acid (v/v; 15 µl). The mixture was then centrifuged for 10 min at 4°C, 13,000 *g*. The supernatants were collected and filtered using a 0.45 µm cellulose acetate membrane filter (Cosmonice Filter W; Nacalai Tesque). The samples were injected into a SIL‐30AC autosampler (Shimadzu). Two serial organic columns (Shim‐pack SCR‐102H, Shimadzu) with a guard column (SCR‐ 102HG; Shimadzu) were used to separate the SCFAs, (acetic acid, butyrate acid, propionic acid, and isobutyric acid). The column conditions were set at 50°C with an isocratic elution (0.8 ml/min) of 5 mmol/L p‐toluene sulfonic acid aqueous solution using a solvent delivery pump (LC‐30AD; Shimadzu). SCFAs were detected using an electronic conductivity detector (CDD‐10Avp, Shimadzu) following postcolumn dissociation (0.8 ml/min) with 5 mmol/L p‐toluene sulfonic acid, 20 mmol/L bis‐Tris, and 100 µmol/L EDTA. SCFAs were quantified with a system controller (CBM‐20A; Shimadzu), and the concentrations of acetate, butyrate, propionate, and isobutyrate were expressed in nmol/mg wet matter.

### 2.8 Statistical analysis

All data are presented as mean ± *SEM*. The effects of diet and treatment were analyzed by one‐way analysis of variance (ANOVA) following Tukey's test. *p* < .05 was considered significant.

## 3 RESULTS

### 3.1 ESM reduces plasma TG and liver TC in HFD‐fed mice

**FIGURE 1:**
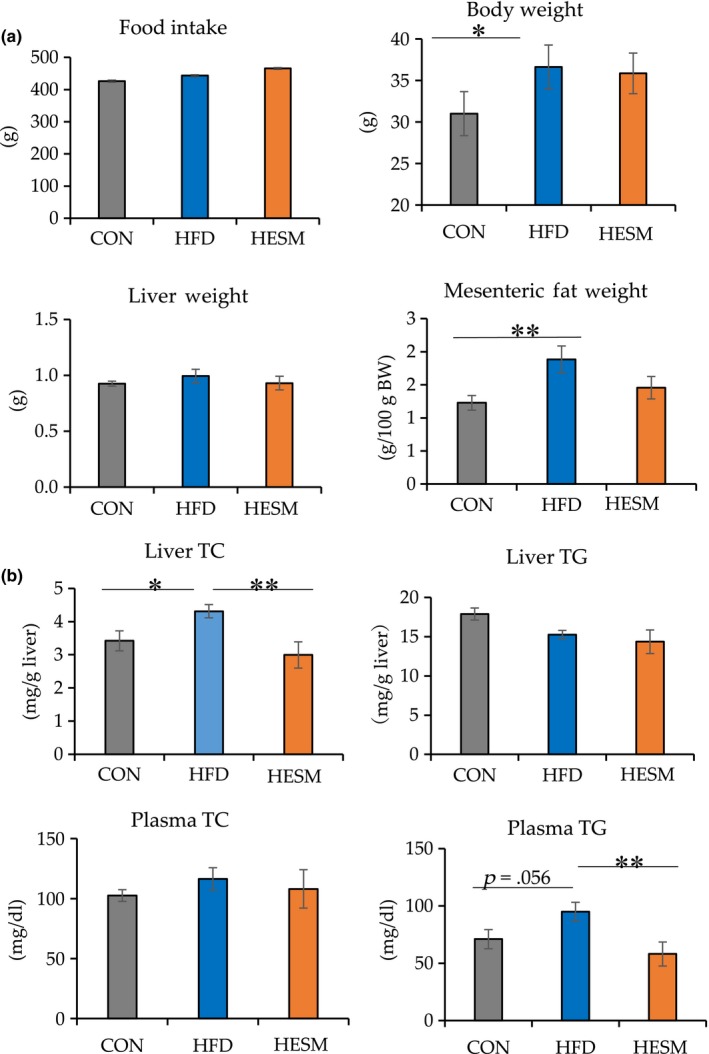
(a) Food intake, body weight, and organ weight and (b) biochemical analysis at 20 weeks of ESM supplementation. Data are presented as means ± *SEM* (*n* = 6–7). **Indicates significant at *p* < .01 and *Indicates significant at *p* < .05 by one‐way ANOVA and Tukey's test. CON, control mice; HESM, high‐fat diet mice + 8% eggshell membrane powder; HFD, high‐fat diet mice; TC, total cholesterol; TG, triglyceride

Figure 1a presents food intake, body weight, and organ weight at 20 weeks of ESM supplementation. Body weight and mesenteric fat weight were increased in HFD mice compared to CON mice. However, no statistically significant differences in food intake, body weight, mesenteric fat weight, and liver weight were observed between the experimental groups following ESM supplementation for 20 weeks. As shown in Figure 1b, biochemical analysis demonstrated that plasma TG content and liver TC were increased by HFD, but significantly suppressed in HESM mice, indicating that ESM improves the lipid profile in HFD mice.

### 3.2 Effects of ESM supplementation on lipid metabolism‐related genes

**FIGURE 2:**
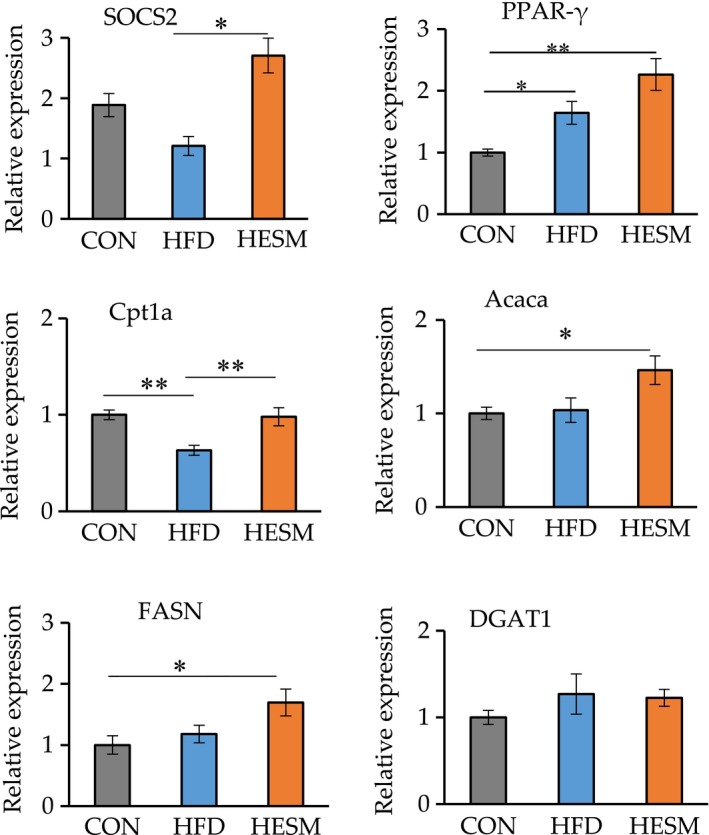
Liver mRNA expression of genes related to lipid metabolism as measured by real‐time RT‐PCR. Data are presented as means ± *SEM* (*n* = 6–7), and **Indicates significant at *p * < .01 and *Indicates significant at *p* < .05 by Tukey's test. CON, control mice; HESM, high‐fat diet mice + 8% eggshell membrane powder; HFD, high‐fat diet mice

We next conducted an analysis of lipid metabolism‐related genes using real‐time RT‐PCR to further elucidate the mechanism underlying the lipid profile improvement in ESM‐supplemented mice. As shown in Figure 2, ESM supplementation upregulated the gene expression level of suppressor of cytokine signaling 2 (*Socs2*), which encodes an important negative regulator of growth hormone (GH; Flores‐Morales, Greenhalgh, Norstedt, & Rico‐Bautista, 2006). SOCS2 inhibits the GH‐induced increase in fatty acid synthase (FASN), peroxisome proliferator‐activated receptor gamma (PPARγ), carnitine palmitoyltransferase 1A (CPT1a), and acetyl‐coenzyme A carboxylase alpha (ACACA; Yang, Sun, Sun, & Qi, 2012). Hence, based on these *Socs2* results, we examined the expression level of genes involved in lipid metabolism. The results also showed that ESM upregulated the expression of *Cpt1a*, encoding an important protein in fatty acid oxidation. The expression level of *Pparγ*, which is upstream of CPT1, tended to increase in the HESM group. However, the expression levels of *Acaca* and *Fasn*, which are associated with lipogenesis, were significantly increased in the HESM group compared with the CON group, while no significant change was observed for diacylglycerol O‐acyltransferase 1 (*Dgat1*), a key gene in TG synthesis.

### 3.3 ESM modulates gut microbiota composition and community structure in HFD‐fed mice

**FIGURE 3:**
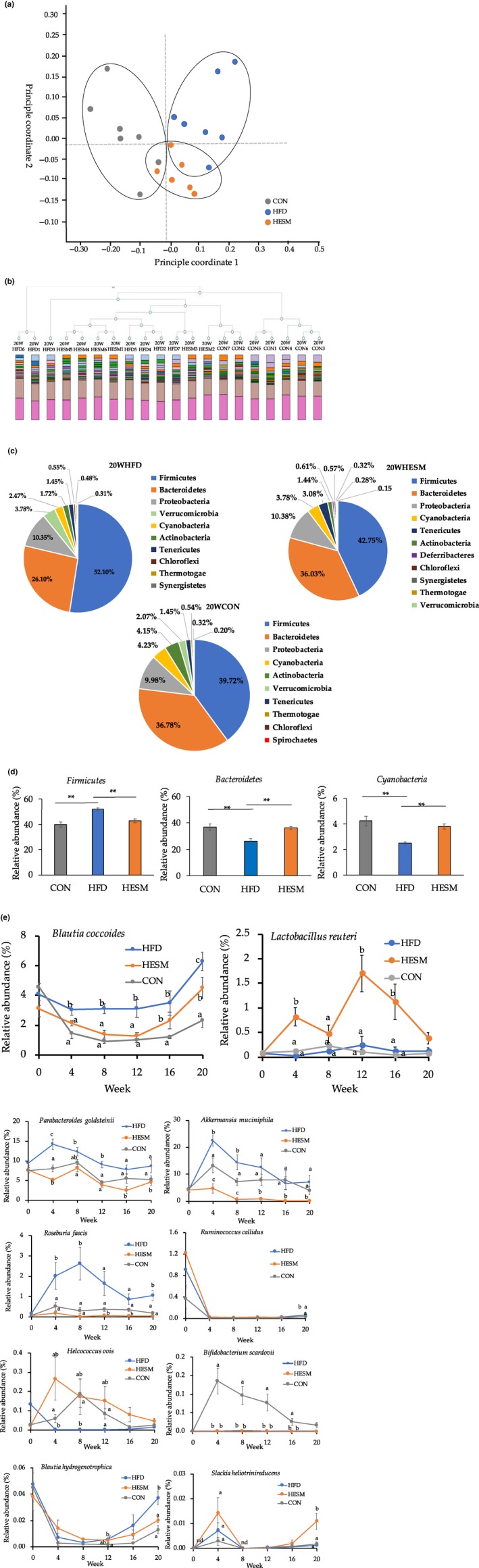
(a) Principal coordinate analysis (PCoA) measurement at the genus level at 20 weeks of ESM supplementation. (b) Hierarchical clustering dendrogram analysis. (c) Taxonomic distribution of microbial community at the phylum level after 20 weeks of ESM supplementation. (d) Relative abundance of obesity‐related Firmicutes, Bacteroidetes, and Cyanobacteria at the phylum level. (e) Trend of obesity‐related microbiota in HFD, HESM, and CON mice from 0 to 20 weeks of ESM supplementation. The results are presented as mean ± *SEM* (*n* = 6–7). **Indicates significant at *p* < .01 and different letters (a, b, c) indicate significant at *p* < .05 by one‐way ANOVA and Tukey's test. CON, control mice; HESM, high‐fat diet mice + 8% eggshell membrane powder; HFD, high‐fat diet mice; nd, not detected

An evaluation of differences in the distribution of microbial composition, up to a fixed taxonomic level, was conducted using PCoA. Figure 3a displays the PCoA results at the genus level after 20 weeks of ESM supplementation in HFD‐fed mice; clear differences were observed between the CON, HFD, and HESM groups. Hierarchical clustering dendrogram analysis demonstrated that the gut microbiota composition of the HESM group was closer to the HFD group than the CON group, as shown in Figure 3b. Figure 3c shows that the predominant phyla in the HFD, HESM, and CON groups were Firmicutes and Bacteroidetes. Figure 3d provides the relative abundance of the obesity‐related Firmicutes, Bacteroidetes, and Cyanobacteria at the phylum level. The relative abundance of Firmicutes was significantly higher in the HFD group (52.10%) compared with the CON (39.72%, *p* = .0007) and HESM (42.75%, *p* = .0004) groups. Notably, ESM supplementation prevented the HFD‐induced decrease in Bacteroidetes (HFD; 26.10%; HESM 36.03%; *p* = .0079) and Cyanobacteria (HFD: 2.47%; HESM; 3.78%; *p* = .0024).

We next examined the changes in the relative abundance of microbiota at 4‐week intervals during the intervention period (Figure 3e). The results demonstrated that 10 bacteria showed a tendency to change during weeks 0–16. The relative abundance of obesity‐related bacterium, *Blautia coccoides*, decreased in the HESM group during weeks 0–12, but displayed an increasing trend at week 16. At week 20, the HESM group showed lower abundance of *B. coccoides* than the HFD group. The relative abundance of the anti‐obesity bacterium, *Lactobacillus reuteri*, remained higher in HESM mice than in HFD and CON mice throughout the intervention period. In addition, the relative abundance of the obesity‐related harmful bacterium, *Parabacteroides goldsteinii*, and pro‐inflammatory *Roseburia faecis* remained lower in the HESM group than in the HFD and CON groups during weeks 4–20.

Our findings also revealed that 89 bacterial communities at the species level were significantly altered, while 65 bacterial communities showed a tendency to change following ESM supplementation (Table S3). The relative abundance ratio of bacterial species associated with obesity, inflammation, diabetes, and oxidative stress are listed in Table 1. We observed that the relative abundance ratio of obesity‐related bacterium, *B. coccoides*, decreased from 2.7027 in HFD versus CON (*p* = .0003) to 0.7150 in HESM versus HFD (*p* = .0840). In contrast, the relative abundance ratio of the anti‐obesity bacterium *L. reuteri* showed an increasing trend in the HESM versus HFD group (3.4053, *p* = .0827). However, *P. goldsteinii* showed a decreasing trend from 1.6424 in HFD versus CON (*p* = .1111) to 0.5288 in HESM versus HFD (*p* = .0608). In addition, we observed that the anti‐obesity bacterium, *Akkermansia muciniphila*, was reduced in the HESM versus HFD group (0.0274, *p* = .0382).

**TABLE 1:** Relative abundance ratio of bacteria after 20 weeks of ESM supplementation

Roles	Species	20 Weeks			
		HFD versus CON		HESM versus HFD	
		Ratio	*p*‐value	Ratio	*p*‐value
Obesity‐related bacteria	*Blautia coccoides*	2.7027	.0003	0.7150	.0840
	*Lactobacillus reuteri*	1.6430	.5999	3.4053	.0827
	*Parabacteroides goldsteinii*	1.6424	.1111	0.5288	.0608
	*Akkermansia muciniphila*	1.8095	.3165	0.0274	.0382
Inflammation‐related bacteria	*Roseburia faecis*	5.7825	.0143	0.0405	.0075
	*Ruminococcus callidus*	1.9611	.0011	0.5014	.0135
	*Helcococcus ovis*	0.5961	.1609	2.9279	.0557
	*Aerococcus christensenii*	1.0406	.9403	3.2613	.0854
	*Bifidobacterium scardovii*	0.0359	.0612	0.0000	.0789
	*Capnocytophaga cynodegmi*	4.7996	.0972	0.0000	.0375
	*Desulfovibrio fairfieldensis*	0.0000	.1407	0.0228/0.0000	.0745
	*Blautia hydrogenotrophica*	2.8189	.0032	0.5484	.0187
Diabetes‐related bacteria	*Slackia heliotrinireducens*	0.6799	.5259	8.5204	.0320
Oxidative stress‐related bacteria	*Bifidobacterium thermophilum*	0.1047	.1076	0.0000	.0961

Note

Firmicutes: *Blautia coccoides*,* Lactobacillus reuteri*,* Roseburia faecis*,* Ruminococcus callidus*,* Helcococcus ovis*,* Aerococcus christensenii*,* Blautia hydrogenotrophica*; Actinobacteria: *Bifidobacterium thermophilum*, *Slackia heliotrinireducens*,* Bifidobacterium scardovii*; Bacteroidetes: *Parabacteroides goldsteinii*,* Capnocytophaga cynodegmi*; Verrucomicrobia: *Akkermansia muciniphila*; Proteobacteria: *Desulfovibrio fairfieldensis*.

Abbreviations: CON, control mice; HESM, high‐fat diet mice + 8% eggshell membrane powder; HFD, high‐fat diet mice.

As shown in Table 1, eight bacterial species were found to be related to the inflammatory response; *R. faecis*, *Ruminococcus callidus*, *Blautia hydrogenotrophica*, and *Slackia heliotrinireducens* were significantly altered following ESM supplementation. The relative abundance ratio of *R. faecis* was fivefold lower in HESM versus HFD (0.0405, *p* = .0075) than in HFD versus CON (5.7825, *p* = .0143). Similarly, *R. callidus* was significantly decreased in the HESM versus HFD group (0.5014, *p* = .0135) compared with HFD versus CON (1.9611, *p* = .0011). Additionally, *B. hydrogenotrophica* decreased from 2.8189 in HFD versus CON (*p* = .0032) to 0.5484 in HESM versus HFD (*p* = .0187). In contrast, the diabetes‐related bacterium, *S. heliotrinireducens*, was significantly increased in the HESM versus HFD group (8.5204, *p* = .0320).

### 3.4 Correlation between gut microbiota and cecal SCFA levels

**FIGURE 4:**
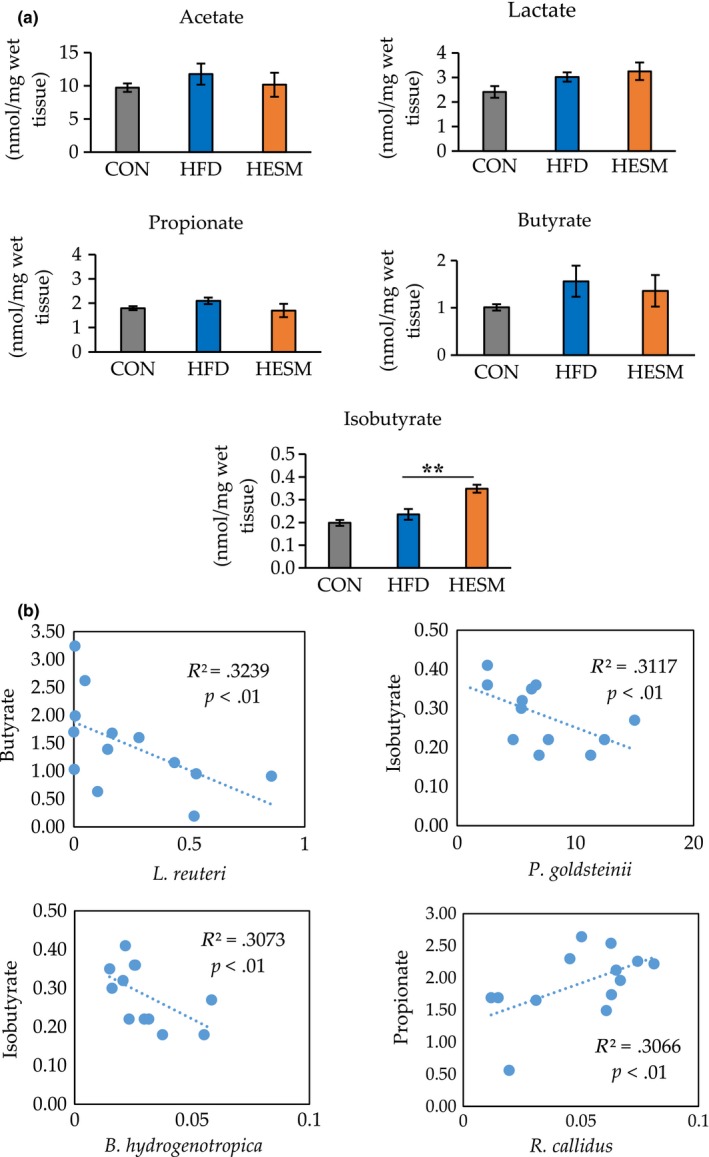
(a) Analysis of cecal SCFA levels in CON, HFD, and HESM mice at 20 weeks of ESM supplementation. (b) Correlations between the relative abundance of bacteria and SCFA levels in HFD and HESM mice. Data are presented as means ± *SEM* (*n* = 6–7), and the significance level was set at *p* < .01 by Tukey's test. CON, control mice; HESM, high‐fat diet mice + 8% eggshell membrane powder; HFD, high‐fat diet mice

As decreases in *Ruminococcus* and *Roseburia* abundance and increased *L. reuteri* are related to the production of SCFAs in the human gut (Cremon et al., 2018), we determined the cecal SCFA levels in CON, HFD, and HESM mice (Figure 4a). Cecal levels of acetate, lactate, butyrate, and propionate did not significantly differ in the CON, HFD, and HESM groups, indicating that the HFD and ESM intervention did not alter the excretion of these SCFAs. However, the branched‐chain fatty acid isobutyrate was significantly increased in HESM compared with the CON and HFD groups.

The results of the correlation analysis are presented in Figure 4b. We observed a weak positive correlation between the abundance of *R. callidus* and propionate concentration (*R* ^2^ = .3066, *p* < .01). In contrast, we found a negative correlation between the abundance of *L. reuteri* and butyrate concentration (*R* ^2^ = .3239, *p* < .01), between the abundance of *P. goldsteinii* and isobutyrate concentration (*R* ^2^ = .3117, *p* < .01), and between the abundance of *B. hydrogenotrophica* and isobutyrate concentration (*R* ^2^ = .3073, *p* < .01).

## 4 DISCUSSION

The present study investigated the ability of ESM to improve obesity‐related complications in HFD‐induced obese mice. Administration of ESM for 20 weeks reduced plasma TG and liver TC, metabolic parameters related to obesity (Hoving et al., 2018). Subsequent experiments showed that ESM supplementation enhanced *Socs2* and *Cpt1a* expression. SOCS2 is thought to be negatively associated with enhanced TG secretion from the liver and insulin resistance (Zadjali et al., 2012), while CPT1 plays an important role in enhancing fatty acid oxidation. Therefore, these results indicate that ESM improves lipid metabolism in HFD‐fed mice possibly via the inhibition of TG secretion and the activation of fatty acid oxidation. However, the expression of *Acaca*, *Fasn* (hepatic genes involved in lipogenesis), and *Dgat1* (involved in the final step of TG synthesis) was not decreased, despite lower plasma TG. Previous studies have reported the reverse changes between TG clearance and lipogenesis (Chen et al., 2019; Wu et al., 2015), as the expression of these gene is regulated by PPARγ (Kersten, 2014). However, our data showed an increase in both parameters, suggesting that lipogenesis‐related genes are regulated by a different mechanism in ESM‐fed mice. In contrast to the lipogenesis parameters, ESM did not influence the mRNA level of *Dgat1*, which encodes an enzyme necessary for TG synthesis; thus, ESM may promote lipogenesis, but not TG synthesis, in the hepatic tissue of mice. Collectively, our data suggest that lipid metabolism might be accelerated by dietary ESM to increase lipogenesis and TG clearance rates, thereby reducing TG content in the plasma. However, further studies are necessary to investigate this possibility.

This study also aimed to investigate the changes in microbial composition in ESM‐fed mice, as recent studies have suggested that changes in composition are strongly linked to obesity (Sivamaruthi, Kesika, Suganthy, & Chaiyasut, 2019; Turnbaugh et al., 2006) and our previous study indicated that ESM can attenuate microbial dysbiosis in an animal model of inflammatory bowel disease (Jia et al., 2017). The PCoA results revealed a clear, distinct microbial composition in the HESM group compared with the HFD group, indicating the potency of ESM supplementation in attenuating microbial dysbiosis caused by HFD. Our analysis at the phylum level found that ESM supplementation reduced Firmicutes, but increased Bacteroidetes and Cyanobacteria abundance. It has been reported that the phylum Firmicutes is negatively correlated with LDL‐cholesterol (Liu et al., 2018). Additionally, increased Bacteroidetes abundance has been implicated in the reduction of TG levels (Caspi et al., 2016), while increased Cyanobacteria abundance has been observed during prebiotic treatment (Everard et al., 2014).

Moreover, our examination at the species level revealed that the relative abundance of the Firmicutes species, *B. coccoides*, was consistently reduced in the ESM‐supplemented group from week 4 to 20. Our results contradict a previous study examining rosemary extract rich in carnosic acid that demonstrated increased *B. coccoides* abundance in parallel with plasma lipid reduction in obese rats (Romo‐Vaquero et al., 2014). The reduced level of liver TC in the present study might be due to an increase in free bile acid concentration in ESM‐supplemented mice. Previous research has shown that free bile acids have bactericidal effects on *B. coccoides*, hence reducing its relative abundance (Watanabe, Fukiya, & Yokota, 2017). Interestingly, ESM supplementation increased *L. reuteri*, an anti‐obesity bacterium associated with high levels of bile acids in the blood (Choi et al., 2016)*. Lactobacillus reuteri* has been shown to ameliorate metabolic abnormalities related to obesity and diabetes mellitus, including TC (Jones, Martoni, & Prakash, 2012), by promoting the production of secondary bile acids (Choi et al., 2016). Prior studies have examined the anti‐obesity effects of *P. goldsteinii* (Neyrinck et al., 2017; Wu et al., 2019), or study showed lower *P. goldsteinii* abundance from week 4 to 20. Similarly, a study on Konjaku flour, which is high in polysaccharides (konjac glucomannan), in obese mice reported a lower abundance of *P. goldsteinii*, in parallel with the amelioration of obesity‐related markers and improvement of intestinal barrier function (Kang et al., 2018).

Obesity is related to chronic inflammation. Our data demonstrated that ESM supplementation may attenuate the inflammatory condition, as evidenced by the reduced *B. hydrogenotrophica* and *R. callidus* abundance at 20 weeks of intervention. Consistent with our findings, high abundance of *Blautia* has been detected in patients with conditions such as nonalcoholic fatty liver diseases and inflammatory bowel syndrome (IBS; Rigsbee et al., 2012). The abundance of another member of the phylum Firmicutes, *Roseburia faecis*, decreased in the ESM group, which is consistent with a prospective cohort study in obese postmenopausal women receiving a very low‐calorie diet. Furthermore, *Ruminococcus* and *Roseburia* species, which were significantly altered in the present study, have been linked to enhanced production of SCFAs (Aleman et al., 2018). For these reasons, we analyzed SCFA levels in the cecal content and their correlation with the relative abundance of bacteria.

We found a positive correlation between the relative abundance of *R. callidus* and the concentration of propionate. In other studies, high butyrate and propionate excretion was observed in obese mice (Turnbaugh et al., 2006), corroborating the idea that a reduction in butyrate and propionate may provide some positive metabolic effects to the host. Additionally, the negative correlations between the abundance of *P. goldsteinii* and *B. hydrogenotrophica* and isobutyrate concentration suggest that a high isobutyrate level may be unfavorable to possibly harmful bacteria (Wang et al., 2018), thus decreasing their abundance.

In order to fully understand the mechanism underlying ESM‐induced decreases in TG and TC content, further investigations are required to identify specific compounds in ESM responsible for the alteration in lipid metabolism genes. For example, a high concentration of calcium is also found in ESM (Bartter et al., 2018), which could potentially increase fat oxidation in obese mice (Gonzalez, Rumbold, & Stevenson, 2012). Moreover, other compounds in ESM, such as collagen, may afford anti‐obesity effects by upregulating CPT1 and preventing hepatic fat accumulation (Woo, Song, Kang, & Noh, 2018). Notably, we have previously reported that the low digestibility of ESM, with similar physiological properties to that of dietary fiber, may enhance intestinal bacteria fermentation (Jia et al., 2017) by providing a nutrient source, and hence might contribute to changes in gut microbiota composition. Therefore, the improvement in lipid profile following ESM supplementation in HFD‐fed mice may be at least partially due to the action of “resistant proteins” in ESM altering microbial composition.

In conclusion, the present study provides evidence that ESM supplementation lowers liver TG and plasma TC possibly by accelerating lipid metabolism genes and suppressing the abundance of obesity‐related harmful bacteria and promoting the growth of anti‐obesity bacteria. These findings may provide a new perspective regarding ESM as a dietary intervention in obesity management.

## CONFLICT OF INTEREST

The authors have declared no conflict of interest.

## ETHICAL APPROVAL

The study protocols were approved by the Animal Care and Use Committee of the University of Tokyo (Approval No. P17‐143). All procedures were conducted according to the relevant rules and regulations.

## Supporting information

Table S1‐S3
