# Supplementary material for: Eggshell membrane powder lowers plasma triglyceride and liver total cholesterol by modulating gut microbiota and accelerating lipid metabolism in high‐fat diet‐fed mice
Source: Food Sci Nutr. 2020 Apr 5;8(5):2512–23. doi: 10.1002/fsn3.1545 (PMC7215208; doi:10.1002/fsn3.1545)
Supplement: Supplementary file 1 — Table S1‐S3 [file FSN3-8-2512-s001.docx]

**Supporting Information**

**Supporting Information Table 1** Diet composition

| **% (w/w)** | **CON** | **HFD** | **HESM** |
| --- | --- | --- | --- |
| Casein | 20.0 | 25.60 | 21.20 |
| L-cystine | 0.3 | 0.36 | 0.31 |
| Maltodextrin 10 | - | 6.00 | 6.00 |
| β-corn starch | 39.75 | - | - |
| α-corn starch | 13.2 | 28.50 | 24.50 |
| Sucrose | 10.0 | 5.50 | 5.50 |
| Soybean oil | 7.0 | 2.00 | 2.00 |
| Lard | - | 20.50 | 20.50 |
| Cellulose | 5.0 | 6.61 | 6.61 |
| Mineral mixture  (AIN93G-Mix) | 3.5 | 3.50 | 3.50 |
| Vitamin mixture  (AIN93G-Mix) | 1.0 | 1.00 | 1.00 |
| Choline bitartrate | 0.25 | 0.25 | 0.25 |
| Tert-Butylhydroquinone | 0.0014 | 0.0045 | 0.0045 |
| Calcium carbonate | - | 0.18 | 0.18 |
| ESM powder | - | - | 8.00 |

**Supporting Information Table 2.** Mouse primer sequences

| **Gene** | **Primer sequence** | |
| --- | --- | --- |
| DGAT1 | Forward | CAACTACGATGCCCCAGTG |
|  | Reverse | AACCCCTCCAGAACTCCAG |
| DGAT2 | Forward | TCTCCAATCTGAGCCTACCC |
|  | Reverse | GCAGCGAGATAACCCAAGAC |
| FASN | Forward | GAGAAGCCATGTGGGGAAG |
|  | Reverse | TGAGCAGGGACAGGACAAG |
| FABP1 | Forward | GCAGAGCCAGGAGAACTTTG |
|  | Reverse | TCTGACACCCCCTTGATGTC |
| Slc27a1 | Forward | TCTCCACTTCCCTCATAGCC |
|  | Reverse | TAGAGCCAACACTAGCCACATC |
| Acaca | Forward | TGGCCTCCACTTTTGCTAC |
|  | Reverse | TATACAAGCCCAGCCCACTC |
| Cpt1a | Forward | CTTCTAATCCCACCCAGTCAG |
|  | Reverse | CCGAGTCTGTCACCAAAATG |
| PPAR | Forward | TGACCCAATGGTTGCTGATTAC |
|  | Reverse | AGGTGGAGATGCAGGTTCTACTTT |

**Supporting Information Table 3. 20**W HESM vs 20W HFD

| **Phylum** | **Species** | **Ratio** | ***p*-value** |
| --- | --- | --- | --- |
| Firmicutes | Lachnospira pectinoschiza | 0.5312 | 0.0128 |
|  | Anaerobranca zavarzinii | 0.0445 | 0.0026 |
|  | Coprobacillus cateniformis | 0.0962 | 0.0080 |
|  | Peptococcus niger | 0.2581 | 0.0222 |
|  | Clostridium alkalicellulosi | 1.7058 | 0.0072 |
|  | Peptoniphilus coxii | 0.7003 | 0.0131 |
|  | Blautia hansenii | 18.4035 | 0.0948 |
|  | Ammonifex thiophilus | 0.0264 | 0.0037 |
|  | Anaerofilum agile | 2.3969 | 0.0175 |
|  | Dethiobacter alkaliphilus | 0.3988 | 0.0016 |
|  | Clostridium proteolyticus | 0.7796 | 0.0029 |
|  | Lactobacillus antri | 3.0958 | 0.0780 |
|  | Sporotomaculum syntrophicum | 0.7202 | 0.0196 |
|  | Thermoanaerobacter thermocopriae | 0.7415 | 0.0846 |
|  | Halanaerobacter chitinivorans | 0.1673 | 0.0008 |
|  | Streptococcus orisratti | 1.6895 | 0.0084 |
|  | Clostridium caenicola | 1.6137 | 0.0042 |
|  | Acholeplasma granularum | 5.0356 | 0.0084 |
|  | Halanaerobium fermentans | 5.6284 | 0.0033 |
|  | Desulfotomaculum halophilum | 0.4332 | 0.0099 |
|  | Heliobacterium sulfidophilum | 0.0120 | 0.0028 |
|  | Clostridium papyrosolvens | 0.0583 | 0.0995 |
|  | Clostridium cavendishii | 0.1077 | 0.0255 |
|  | Heliobacterium gestii | 0.0000 | 0.0032 |
|  | Alkaliphilus peptidifermentans | 1.9743 | 0.0585 |
|  | Alkalibacterium subtropicum | 0.4054 | 0.0190 |
|  | Desulfotomaculum australicum | 0.0000 | 0.0196 |
|  | Peptoniphilus indolicus | 0.2505 | 0.0332 |
|  | Planococcus columbae | 0.3502 | 0.0873 |
|  | Fusibacter paucivorans | 0.1808 | 0.0229 |
|  | Desulfotomaculum thermoacetoxidans | 0.1347 | 0.0107 |
|  | Paenibacillus donghaensis | 0.0000 | 0.0044 |
|  | Paenibacillus apiaries | 0.2336 | 0.0982 |
|  | Lactobacillus apis | 0.0000 | 0.0959 |
|  | Megasphaera hominis | 0.2440 | 0.0687 |
|  | Bacillus drentensis | 4.7498 | 0.045 |
|  | Pelotomaculum thermopropionicum | 14.2731 | 0.0000 |
|  | Pediococcus siamensis | 0.2100 | 0.0888 |
|  | Lactobacillus lindneri | 3.6318 | 0.0586 |
|  | Paenibacillus popilliae | 0.0000 | 0.0505 |
|  | Coprothermobacter platensis | 0.0000 | 0.0981 |
|  | Anaerobranca californiensis | 0.0000 | 0.0800 |
|  | Pediococcus stilesii |  | 0.0273 |
|  | Weissella salipiscis | 0.0000 | 0.0896 |
|  | Lactobacillus panis |  | 0.0810 |
|  | Desulfosporosinus acidiphilus | 0.0000 | 0.0876 |
|  | Streptococcus troglodytae | 0.0000 | 0.0789 |
|  | Clostridium clariflavum |  | 0.0810 |
| Bacteroidetes | Dysgonomonas hofstadii | 1.6052 | 0.0687 |
|  | Porphyromonas canis | 1.5837 | 0.0053 |
|  | Bacteroides salanitronis | 1.8034 | 0.0397 |
|  | Runella limosa | 45.1695 | 0.0010 |
|  | Bacteroides gallinarum | 1.4042 | 0.0888 |
|  | Bacteroides helcogenes | 2.3597 | 0.0917 |
|  | Zhouia amylolytica | 1.5972 | 0.0100 |
|  | Bacteroides oleiciplenus | 0.6224 | 0.0998 |
|  | Polaribacter butkevichii | 5.4962 | 0.0066 |
|  | Rhodothermus clarus | 0.6813 | 0.0584 |
|  | Sphingobacterium kitahiroshimense | 2.8120 | 0.0634 |
|  | Bacteroides heparinolyticus | 10.4441 | 0.0107 |
|  | Dysgonomonas gadei | 2.8173 | 0.0743 |
|  | Gillisia limnaea |  | 0.0790 |
|  | Sphingobacterium daejeonense | 4.8005 | 0.0674 |
|  | Niabella aurantiaca | 0.1761 | 0.0977 |
|  | Psychroserpens burtonensis |  | 0.0790 |
|  | Psychroflexus lacisalsi |  | 0.0792 |
|  | Olivibacter terrae | 2.1779 | 0.0759 |
|  | Riemerella columbina | 1.6075 | 0.0421 |
| Actinobacteria | Eggerthella sinensis | 5.3207 | 0.0029 |
|  | Kribbella ginsengisoli | 9.2648 | 0.0558 |
|  | Ferrimicrobium acidiphilum | 0.6249 | 0.0024 |
|  | Nocardia alba | 0.6480 | 0.0158 |
|  | Actinobaculum suis | 0.5914 | 0.0184 |
|  | Amycolatopsis tolypomycina | 0.5377 | 0.0041 |
|  | Atopobium fossor | 0.7195 | 0.0950 |
|  | Actinocorallia cavernae | 2.1041 | 0.0460 |
|  | Euzebya tangerine | 0.6336 | 0.0127 |
|  | Brevibacterium album | 0.6874 | 0.0513 |
|  | Streptomyces danangensis | 0.7320 | 0.0249 |
|  | Actinomadura Latina | 0.4168 | 0.0027 |
|  | Rubrobacter taiwanensis | 0.6011 | 0.0953 |
|  | Acidimicrobium ferrooxidans | 0.0726 | 0.0106 |
|  | Gordonia rhizosphere | 0.2620 | 0.0699 |
|  | Demequina aurantiaca | 11.3933 | 0.0492 |
|  | Amycolatopsis nigrescens | 0.2709 | 0.0583 |
|  | Saccharopolyspora salina | 0.1898 | 0.0169 |
|  | Lentzea californiensis | 8.0409 | 0.0680 |
|  | Actinocorallia aurantiaca | 0.0000 | 0.0309 |
|  | Microbacterium xinjiangensis | 0.0000 | 0.0876 |
| Verrucomicrobia | Prosthecobacter fluviatilis | 0.0303 | 0.0367 |
|  | Luteolibacter algae | 0.0691 | 0.0345 |
|  | Rubritalea tangerine | 0.0299 | 0.0284 |
|  | Pelagicoccus croceus | 0.2635 | 0.0236 |
| Proteobacteria | Salinivibrio budaii | 0.6693 | 0.0154 |
|  | Pelobacter carbinolicus | 4.6457 | 0.0076 |
|  | Candidatus Blochmannia castaneus | 1.5168 | 0.0251 |
|  | Marinobacter arcticus | 1.5350 | 0.0548 |
|  | Ehrlichia ovina | 0.7308 | 0.0404 |
|  | Desulfonauticus autotrophicus | 2.3449 | 0.0043 |
|  | Halomonas neptunia | 1.5958 | 0.0115 |
|  | Sulfurimonas paralvinellae | 0.6590 | 0.0238 |
|  | Moritella japonica | 1.5238 | 0.0730 |
|  | Desulfovibrio butyratiphilus | 52.7361 | 0.0970 |
|  | Sphingomonas insulae | 0.5304 | 0.0190 |
|  | Rhodobacter apigmentum | 1.3503 | 0.0284 |
|  | Desulfomonile tiedjei | 0.2670 | 0.0292 |
|  | Inquilinus ginsengisoli | 5.1019 | 0.0725 |
|  | Gallibacterium melopsittaci | 14.3891 | 0.0548 |
|  | Rhodobacter gluconicum | 0.1314 | 0.0110 |
|  | Sphingomonas melonis | 0.5754 | 0.0866 |
|  | Salinivibrio siamensis | 0.4068 | 0.0116 |
|  | Mannheimia caviae | 2.7226 | 0.0082 |
|  | Desulfovibrio cuneatus |  | 0.0479 |
|  | Hyphomonas hirschiana | 0.0349 | 0.0871 |
|  | Candidatus Liberibacter solanacearum | 0.1151 | 0.0172 |
|  | Desulfovibrio frigidus |  | 0.0881 |
|  | Acinetobacter parvus | 0.1984 | 0.0448 |
|  | Roseomonas lacus | 0.3080 | 0.0497 |
|  | Marinobacter squalenivorans | 0.1376 | 0.0106 |
|  | Klebsiella granulomatis |  | 0.0310 |
|  | Azospirillum halopraeferens |  | 0.0383 |
|  | Erwinia psidii |  | 0.0818 |
|  | Serratia entomophila | 2.5656 | 0.0613 |
| Tenericutes | Anaeroplasma abactoclasticum | 1.5610 | 0.0280 |
|  | Anaeroplasma varium | 2.5937 | 0.0823 |
|  | Candidatus Phytoplasma graminis | 5.0468 | 0.0959 |
|  | Mesoplasma entomophilum | 2.3675 | 0.0987 |
| Thermotogae | Fervidobacterium gondwanense | 3.3158 | 0.0494 |
|  | Marinitoga piezophile | 0.0314 | 0.0049 |
|  | Fervidobacterium pennivorans | 0.3780 | 0.0935 |
|  | Fervidobacterium islandicum | 0.5321 | 0.0230 |
|  | Thermosipho ferriphilus | 0.4350 | 0.0137 |
|  | Geotoga petraea | 0.0000 | 0.0618 |
| Spirochaetes | Brachyspira ibaraki | 5.3869 | 0.0138 |
|  | Leptospira licerasiae | 0.6708 | 0.0930 |
| Cyanobacteria | Calothrix parietina | 1.3994 | 0.0189 |
| Chloroflexi | Thermobaculum terrenum | 0.4865 | 0.0029 |
| Planctomycetes | Candidatus Scalindua brodae | 0.0320 | 0.0559 |
| Deinococcus-Thermus | Deinococcus soli | 0.4623 | 0.0257 |
|  | Acholeplasma ales | 4.4907 | 0.0981 |
